# Fragility Fracture Network in the Nordic Orthopaedic Federation countries: the role of orthopedic surgeons

**DOI:** 10.2340/17453674.2026.45296

**Published:** 2026-01-23

**Authors:** Alma B PEDERSEN, Frede FRIHAGEN, Lene B SOLBERG, Peter VAN DEN BERG, Marsha VAN OOSTWAARD, Johanna RUNDGREN, Karl-Åke JANSSON, Henrik PALM

**Affiliations:** 1 Department of Clinical Epidemiology, Aarhus University Hospital, and Department of Clinical Medicine, Aarhus University, Aarhus, Denmark; 2 Department of Orthopaedics, Østfold Hospital Trust, Norway and Institute of Clinical Medicine, University of Oslo, Norway; 3 Division of Orthopaedic Surgery, Oslo University Hospital Ullevål, Norway; 4 Fracture Liaison Service, Department of Orthopaedics and Trauma Surgery, Reinier de Graaf Gasthuis, Delft, The Netherlands; 5 Department of Internal Medicine, VieCuri Medical Center, Venlo, and NUTRIM Institute of Nutrition and Translational Research in Metabolism, Maastricht University Medical Center, Maastricht, The Netherlands; 6 Department of Orthopaedics, Södersjukhuset, Stockholm and Department of Clinical Science and Education, Södersjukhuset, Karolinska Institutet, Stockholm, Sweden; 7 Department of Orthopaedic Surgery, Copenhagen University Hospital, Bispebjerg, Copenhagen, Denmark

## Fragility fractures: urgency and complexity

The global burden of osteoporosis-related fragility fractures has been increasing over recent decades, primarily due to an ageing population [1,2]. Hip fractures, the most severe type of fragility fracture, are projected to nearly double by 2050 compared with 2018 levels [3]. These fractures impose a substantial burden not only on patients but also on their families and societies with rising healthcare expenses [1]. Patients with fragility fractures are typically frail and multimorbid, which complicates both their treatment and recovery [4]. They are at increased risk of surgical complications, delirium, infections, opioid dependence, failure to regain prefracture mobility status, and elevated mortality [5]. A fragility fracture substantially increases the risk of falls and new fractures within the first 2 years [6] with even higher excess mortality [7]. Thus, secondary fracture prevention is urgent. Reflecting on this, the landmark paper Global call to action to improve the care of individuals with fragility fractures was published in 2018 [8]. It was endorsed by 81 professional organizations worldwide—including several orthopedic associations and the Fragility Fracture Network (FFN).

## The role of orthopedic surgeons in the multidisciplinary strategy (Figure 1)

To address the growing burden of fragility fracture patients and improve their care, there is an urgent need for an evidence-based multidisciplinary strategy [8-10] that can bridge the entire clinical course, from the initial injury through hospital treatment to community-based recovery. The multidisciplinary strategy includes, but is not limited to, orthogeriatric acute care [11], integrated rehabilitation [12], and secondary fracture prevention [13,14]. Orthopedic surgeons play a key role as leaders or facilitators in this strategy through:

- Determining the most appropriate treatment, be this non-surgical or surgical, considering fracture type, surgical method and implant, timing of surgery, and weightbearing regime.
- Identifying and considering patient challenges related to their medical, cognitive, physical, and social status when deciding on treatment.
- Optimizing acute fracture care by assessing and managing pain, delirium, malnutrition, infections, venous thromboembolism, need for fluid resuscitation, and promoting early mobilization.
- Initiating secondary fall and fracture prevention by assessing the cause of falling and the risk of future falls, investigating history and risk of underlying osteoporosis, and ensuring follow-up within a local Fracture Liaison Service (FLS).
- Monitoring surgical outcomes like mortality, bleeding, wound infection, fracture healing, osteosynthesis/implant failure, and reoperation.
- Enhancing the focus on functional recovery, quality of life, and return to home—outcomes increasingly recognized as important by patients and their caregivers.
- Ensuring clear communication of patient needs to general practitioners, rehabilitation centers, and family/caregivers following discharge.
- Promoting evidence-based care of fragility fracture patients through high-quality clinical research, education, and translation of knowledge into clinical practice.

**Figure 1:**
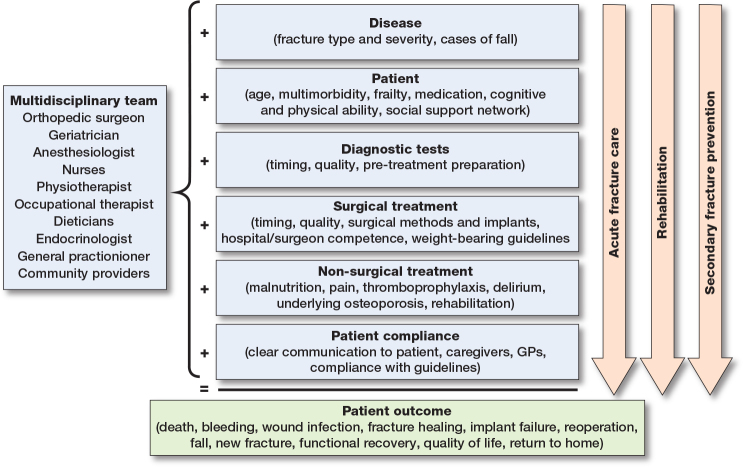
Role of the orthopedic surgeon and other members of the multidisciplinary orthogeriatric team in evaluating the entire clinical course of patients with fragility fractures.

## Introduction to Global FFN

The FFN was founded in Europe in 2011 and is one of only a few global organizations with a truly interdisciplinary membership. The first international FFN congress took place in 2012. The FFN now has about 14,000 members, of which 23% are orthopedic surgeons, from more than 100 countries covering all continents. The FFN vision is a world in which every individual who sustains a fragility fracture achieves optimal recovery of independent function and quality of life, without experiencing subsequent fractures. The FFN mission is to optimize globally the multidisciplinary management of the patient with a fragility fracture. It operates on 4 pillars: (i) acute multidisciplinary care, (ii) rehabilitation, (iii) secondary prevention, and (iv) policy change. The pillars are built on close collaboration among a united team of professionals including orthopedic surgeons, geriatricians, anesthesiologists, nurses, physiotherapists, occupational therapists, dietitians, and osteoporosis specialists, with outreach to general practitioners and community-based healthcare providers. The FFN offers several learning activities through six Special Interest Groups, mentorships, and focused educational webinars, guidelines, and open access books [15-17]. The FFN also provides a unique platform for interdisciplinary research collaboration, particularly in areas that extend beyond the scope of a single specialty [13,18-22]. The policy pillar emphasizes the establishment of multidisciplinary regional and national FFN organizations and alliances aimed at driving policy reforms that support the implementation of the first 3 pillars. This approach acknowledges the necessity of adapting strategies to the unique and diverse structures of individual healthcare systems. Still, what proves effective in one country may be transferable to another. For example, some countries have implemented evidence-based clinical care standards such as comprehensive geriatric assessment, early surgery, early mobilization, delirium screening, bone health, and falls assessment through national hip fracture registries and audits [18]. Adherence to clinical care standards is associated with improved patient survival [23]. These initiatives can serve as a valuable source of inspiration for other countries, especially within geographically and culturally aligned regions such as the Nordic area.

## Introduction to established FFN organizations in Nordic Orthopaedic Federation countries

The FFN Norway was established in 2018 and currently comprises approximately 700 members, of which 17% are orthopedic surgeons. FFN Norway has initiated several activities including hosting annual meetings, the development of interdisciplinary national hip fracture guidelines, the revision of physiotherapy and early rehabilitation guidelines, and the publication of guidelines for secondary prevention [24]. The FFN Netherlands was established in 2023 and currently focuses on defining and strengthening the roles of nurses and nurse practitioners through FLS. The FFN Denmark was established in October 2023 and currently includes 386 members, of which 9% are orthopedic surgeons. Since its inception, FFN Denmark has organized 2 national meetings featuring sessions on various patient care topics and including more than 30 scientific abstracts at each meeting. FFN Denmark is currently leading a multidisciplinary initiative in collaboration with the Danish Osteoporosis Association and 6 medical specialty societies to implement FLSs in all Danish hospitals starting January 2026. An FLS can substantially reduce the risk of new fractures by ensuring systematic assessment, management, and monitoring of osteoporosis and risk of falls [14,24]. The initiative to establish FFN Sweden has come from the orthopedic community. FFN Sweden aims to improve the multidisciplinary care of patients with fragility fractures and fracture prevention. FFN Sweden plans to act in close collaboration with other relevant national associations to increase awareness, organize educational programs, host annual meetings, and take part in updating national best practice guidelines, as well as advocating policy change.

At present (September 2025), there are no established FFN organizations in Estonia, Finland, Iceland, or Lithuania, although several healthcare professionals have been active in the Global FFN.

## Conclusion

Although the FFN organization is relatively new, it represents a vital and rapidly growing network for advancing the care of patients with fragility fractures through multidisciplinary collaboration, education, and implementation of best practices. Due to their central role in fracture management, orthopedic surgeons across the globe should embrace the multidisciplinary FFN framework and join the team. The responsibility and of opportunity for orthopedic surgeons to act as leaders, collaborators, communicators, and researchers within the multidisciplinary team—both within Global FFN and the national FFNs—are crucial in improving outcomes and ensuring patient safety in the care of patients with fragility fractures.

## Disclosures

Complete disclosure of interest forms according to ICMJE are available on the article page, doi: 10.2340/17453674.2026.45296

## Notes

Handling co-editor: Paul Gerdhem

*Acta* thanks Olle Svensson and Per Swärd for help with peer review of this manuscript.
